# Qualification of Non-Halogenated Organic Solvents Applied to Microsphere Manufacturing Process

**DOI:** 10.3390/pharmaceutics12050425

**Published:** 2020-05-06

**Authors:** Hyunjin Shim, Hongkee Sah

**Affiliations:** College of Pharmacy, Ewha Womans University, 52 Ewhayeodaegil, Seodaemun-gu, Seoul 03760, Korea; jinshim@ewhain.net

**Keywords:** poly-d,l-lactide-*co*-glycolide, microspheres, microencapsulation, solvent qualification

## Abstract

As a non-halogenated dispersed solvent, ethyl acetate has been most commonly used for the manufacturing of poly-d,l-lactide-*co*-glycolide (PLGA) microspheres. However, ethyl acetate-based microencapsulation processes face several limitations. This study was aimed at proposing ethyl formate as an alternative. Evaluated in this study was the solvent qualification of ethyl formate and ethyl acetate for microencapsulation of a hydrophobic drug into PLGA microspheres. An oil-in-water emulsion solvent extraction technique was developed to load progesterone into PLGA microspheres. Briefly, right after emulsion droplets were temporarily stabilized, they were subject to primary solvent extraction. Appearing semisolid, embryonic microspheres were completely hardened through subsequent secondary solvent extraction. Changes in process parameters of the preparative technique made it possible to manipulate the properties of emulsion droplets, progesterone behavior, and microsphere quality. Despite the two solvents showing comparable Hansen solubility parameter distances toward PLGA, ethyl formate surpassed ethyl acetate in relation to volatility and water miscibility. These features served as advantages in the microsphere manufacturing process, helping produce PLGA microspheres with better quality in terms of drug crystallization, drug encapsulation efficiency, microsphere size homogeneity, and residual solvent content. The present ethyl formate-based preparative technique could be an attractive method of choice for the production of drug-loaded PLGA microspheres.

## 1. Introduction

There have been wide interests in development of poly-d,l-lactide-*co*-glycolide (PLGA) microspheres as long-acting parenteral depots [1,2,3]. Representative PLGA microsphere products in the marketplace are Arestin, Bydureon, Decapeptyl SR, Lupron Depot, Parlodel LAR, Risperdal Consta, Sandostatin LAR Depot, Triptodur, Vivitrol, and Zilretta. PLGA microspheres are usually manufactured by emulsion-based solvent evaporation/extraction methods. From a scientific viewpoint, a suitable dispersed solvent should meet the following criteria: dissolving power for hydrophobic PLGA polymers; water immiscibility required to make an emulsion; and volatility ensuring easy removal during/after manufacturing. Since methylene chloride fulfills these requirements, the solvent has been overwhelmingly used to prepare PLGA microspheres [3,4,5]. However, the solvent not only causes CNS depression but also is irritating to the skin, eyes, and respiratory tract. Carbon monoxide, a metabolite of methylene chloride, also triggers adverse health effects. Because of these toxicological profiles, the solvent is found in the ATSDR (Agency for Toxic Substances and Disease Registry) 2019 Substance Priority List (#90 out of total 275 hazardous substances). The Department of Health and Human Services (DHHS, USA) also considers methylene chloride a human carcinogen. Chloroform is another solvent that is qualified for an emulsion-based microencapsulation process, and it is frequently used to prepare polymeric microspheres [6,7,8]. This halogenated solvent (#11 in the ATSDR Substance Priority List), however, is more toxic than methylene chloride. The ICH classifies methylene chloride and chloroform as Class 2 solvents [9].

Emerging as a substitute for the above halogenated solvents, ethyl acetate is a dispersed solvent of choice for manufacturing PLGA microspheres [10,11,12]. Ethyl acetate belongs to the ICH Class 3 solvents that are less toxic and more environmentally friendly. However, ethyl acetate-based microencapsulation processes face several drawbacks. For example, the solvent has poor solvation power on PLGA with equal molar ratio of lactide to glycolide, forms fiber-like PLGA agglomerates, and produces deflated microspheres with wrinkles on their surface [13,14,15,16]. Other problems include low drug encapsulation efficiency and poor microsphere production yield. Previously, Sah suggested the possible applicability of ethyl formate to the preparation of PLGA microspheres [17]. However, its qualification as a dispersed solvent for microencapsulation was not thoroughly evaluated at that time. Ethyl formate is listed as an ICH Class 3 solvent that is favored by the pharmaceutical industry. As seen in Table 1, the solvent has a boiling point (52–54 °C) that is much lower than ethyl acetate (77 °C). In addition, the former has better water miscibility, compared to the latter. These properties allow the solvent staying in emulsion droplets to be efficiently extracted and removed from a microsphere suspension. In addition, the solvent’s low boiling point is advantageous when microspheres are subject to drying. Based on these considerations, our study was aimed at evaluating the qualification of two non-halogenated solvents (ethyl formate and ethyl acetate) as dispersed solvents for the preparation of PLGA microspheres. To do so, a solvent extraction microencapsulation technique was developed. Critical parameters related to microsphere formulation and microencapsulation process were identified and assessed in this study. In addition, the major quality attributes of PLGA microspheres prepared using the two solvents were compared to each other. Progesterone was used as a model drug throughout this study.

## 2. Materials and Methods

### 2.1. Materials

Poly-d,l-lactide-*co*-glycolide (PLGA) with a lactide:glycolide ratio of 75:25 (7525DLG 4A; inherent viscosity, 0.4 dL/g in CHCl_3_ at 25 °C) was purchased from Evonik Degussa Corp. (Birmingham, AL, USA). Polyvinyl alcohol (PVA; 88% hydrolyzed, Mw = 25,000 g/mol) was supplied from Polysciences, Inc. (Warrington, PA, USA). Progesterone was purchased from TCI Chemicals (Tokyo, Japan). Ethyl formate (EF) and sodium dodecyl sulfate (SDS) were from Sigma-Aldrich Korea (Seoul, Korea). Ethyl acetate (EA) was purchased from Avantor Performance Materials Korea, Ltd. (Suwon, Korea).

### 2.2. Preparation of Poly-d,l-lactide-co-glycolide (PLGA) Microspheres

PLGA microspheres were prepared as follows. PLGA (250 or 400 mg) and progesterone (70, 100, or 130 mg) were dissolved in 3 mL of a non-halogenated organic solvent (EF or EA). This solution was used as a dispersed phase. Separately, the organic solvent (0, 1, or 2 mL) was dissolved in 25 mL of a 0.5% PVA aqueous solution, which was used as an aqueous continuous phase. The dispersed phase was gently poured onto the continuous phase being stirred at 450 rpm by a digital plate stirrer (model 400 series; VWR Scientific, Radnor, PA, USA). This step took only a few seconds. After 5 min, 50 mL of a 0.1% PVA solution was added to the oil-in-water (o/w) emulsion (this step was carried out to generate semisolid emulsion droplets/embryonic microspheres). In 5 min, the whole emulsion was poured into 100 mL of a 0.1% PVA solution and was subject to continual stirring at 450 rpm for 1 h (this process was termed as the primary solvent extraction step). After a sieve with a pore size of 425 μm was placed on a sieve with a pore size of 25 μm, the microsphere suspension was poured onto the sieve with the larger pore size (this process was defined as the wet sieving step). The microspheres present between the two sieves were redispersed in 100 mL of the fresh 0.1% PVA solution and stirred at 30 °C for 3 h (this treatment was referred to as the secondary solvent extraction step). The resultant microspheres were collected by filtration and vacuum dried overnight at ambient temperature. A microsphere batch with a specific formulation was prepared at least triplicate. Hereinafter, EF microspheres stated for the microspheres prepared by using ethyl formate. EA microspheres represented those prepared by using ethyl acetate. 

### 2.3. Optical Microscopy

At the end of the primary solvent extraction step, aliquots of a microsphere suspension were sampled and placed onto a glass slide. The status of the microsphere suspension was carefully monitored by the Axio Scope A1 microscope (Carl Zeiss Co., Ltd., Seoul, Korea). 

### 2.4. Microsphere Size Distribution

The particle size distribution of a microsphere suspension was determined by the Mastersizer 3000E (Malvern Instruments Ltd., Worcestershire, UK). A microsphere suspension appearing at the end of the primary solvent extraction step, without being subject to wet sieving, was diluted with water to the final volume of 540 mL, and placed inside the particle size analyzer. A volume-based size was reported in text. A span index was also calculated as (D_90%_ − D_10%_)/D_50%_. The D-values were the intercepts for 10%, 50%, and 90% of the cumulative volume distribution. For instance, D_10%_ was the microsphere size at which 10% of the volume distribution was less than this value.

### 2.5. Interfacial Tension Measurement

A drop shape analyzer (model DSA100; Krűss GmbH, Hamburg, Germany) was used to measure EF– or EA–aqueous phase interfacial tension. Each organic solvent served as a bulk liquid phase. Water containing 0 to 7.5% of each organic solvent was put into a needle. The shape of a drop suspended from the needle in the bulk liquid phase was analyzed, and the pendant drop principle was utilized to calculate interfacial tension. Each experiment was repeated at least 5 times, and interfacial tension was expressed as the mean ± standard deviation.

### 2.6. Atomic Force Microscopy (AFM)

An atomic force microscope (model NX-10; Park Systems Corp., Suwon, Korea) was used to measure the surface roughness of EF and EA microspheres produced under various manufacturing conditions. Microspheres were fixed on a carbon tape, and topographical images of their surface were obtained by scanning a tip fixed to a cantilever (OMCL-AC160TS, Olympus Korea Co., Ltd., Seoul, Korea) in a non-contact mode (resonance frequency of 260 kHz, force constant of 26 N/m). Images were processed by using the XEI software program (4.3.4 Build 22, Park Systems Corp.).

### 2.7. Drug Encapsulation Efficiency 

Microsphere samples, accurately weighed, were dissolved in 4 mL of tetrahydrofuran, which was diluted with 16 mL of a methanol–water mixture (8:2). The appearing PLGA precipitates were removed by the Spin-X centrifuge filter with a 0.22 μm pore size. For HPLC analysis, the filtrate was passed through the Luna C18 column (5 μm, 100 Å) by the methanol–water mixture at a flow rate of 0.8 mL/min. Progesterone eluting from the column was detected at 254 nm, and its concentration was determined by a standard calibration curve. The following equation was used to determine progesterone encapsulation efficiency (EE):

Progesterone EE% = 100 × (actual drug loading ÷ theoretical drug loading)(1)

where actual drug loading was (measured progesterone content in microspheres) ÷ (microsphere sample weight), and theoretical drug loading represented (progesterone amount used for microencapsulation) ÷ (the combined amount of PLGA and progesterone used to prepare microspheres).

### 2.8. Scanning Electron Microscopy (SEM)

Microspheres were mounted onto a double adhesive tape attached to a metal stub. The microspheres were sputtered with gold by using a SC7620 sputter coater (VG Microtech, West Sussex, UK). Their morphology was observed by the JSM-5200 scanning electron microscope (Jeol Inc., Tokyo, Japan). 

### 2.9. Differential Scanning Calorimetry (DSC)

A Tzero hermetic lid was put onto a Tzero aluminum pan containing microsphere samples, which was sealed with the Tzero™ press. The Q2000 differential scanning calorimeter (TA Instruments, New Castle, DE, USA) was used to investigate their thermal behavior. Microsphere samples were heated from 30 to 160 °C at 10 °C/min. DSC balance and cell were purged by nitrogen gas at flow rates of 40 and 60 mL/min, respectively.

### 2.10. Thermogravimetric Analysis (TGA)

TGA was performed on microspheres (model Q50 thermogravimetric analyzer; TA Instruments, New Castle, DE, USA). EF or EA microspheres were placed in a platinum sample pan. The system was heated from 30 to 550 °C at a heating rate of 20 °C/min in nitrogen atmosphere with a purge rate of 40 mL/min. The mass reduction observed from 30 to 115 °C was expressed as the residual solvent content in the microsphere sample. 

### 2.11. Gas Chromatography (GC)

EF or EA microspheres were dissolved in 4 mL of tetrahydrofuran. Each sample solution was diluted with 12 mL of methanol. PLGA precipitates were removed by the Spin-X centrifuge filter with a pore size of 0.22 μm. The resulting solution was spiked with an internal standard solution. An aliquot (1 μL) of the samples was injected into the injection port of the gas chromatograph (model GC 2010; Shimadzu Corp., Kyoto, Japan). The vaporized analytes were carried to the ZB-624 column (30 m in length, 0.32 mm in inner diameter) by nitrogen gas at the flow rate of 1.75 mL/min. The analytes eluting from the column were detected by FID. EF or EA concentrations were determined using standard calibration curves. To quantity EF, its standard solutions with known concentrations were prepared by the methanol–tetrahydrofuran mixture. They were then spiked with an EA internal standard solution. In case of determining EA concentrations, its standard solutions were spiked with an EF internal standard solution prior to GC analysis. 

### 2.12. Drug Release Test

Microspheres were prepared by emulsifying a dispersed phase (400 mg of PLGA, 100 mg of progesterone, and 3 mL of EF or EA) in an aqueous phase containing 1 mL of the solvent. Microsphere samples (40 mg) were dispersed in 20 mL of a 0.5% SDS-containing phosphate buffer (20 mM, pH 7.4) at 45 °C. The microsphere suspension was slowly agitated at 20 rpm by using a mixer (model SLRM-3 multimixer; Seoulin Bioscience, Korea). An aliquot (5 mL) of the dissolution medium was sampled at regular time intervals, and the microsphere suspension was replenished with 5 mL of the fresh medium. Progesterone concentrations were analyzed by the HPLC method described earlier.

## 3. Results

**Figure 1:**
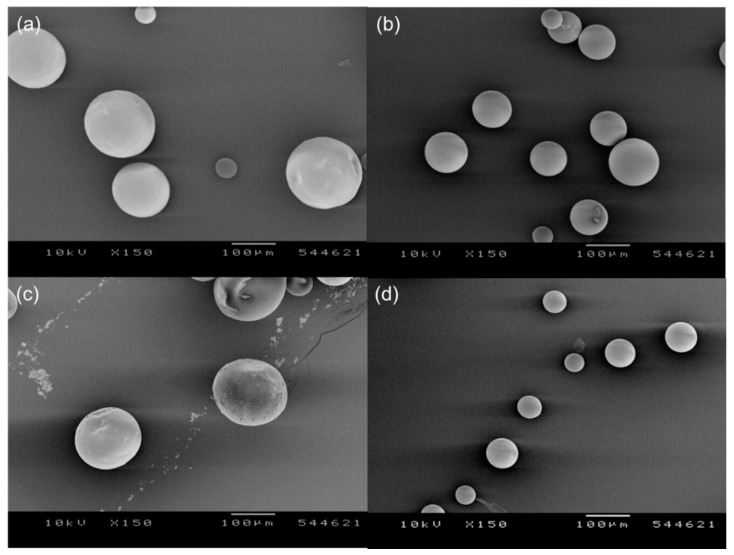
SEM micrographs demonstrating the effect of solvent type and its pre-dissolved amount upon microsphere morphology. Microspheres were prepared using either (**a**,**b**) ethyl formate (EF) or (**c**,**d**) ethyl acetate (EA). Prior to emulsification, an aqueous continuous phase was dissolved with (left) 0 or (right) 2 mL of each solvent. The bar size is 100 μm.

**Figure 2:**
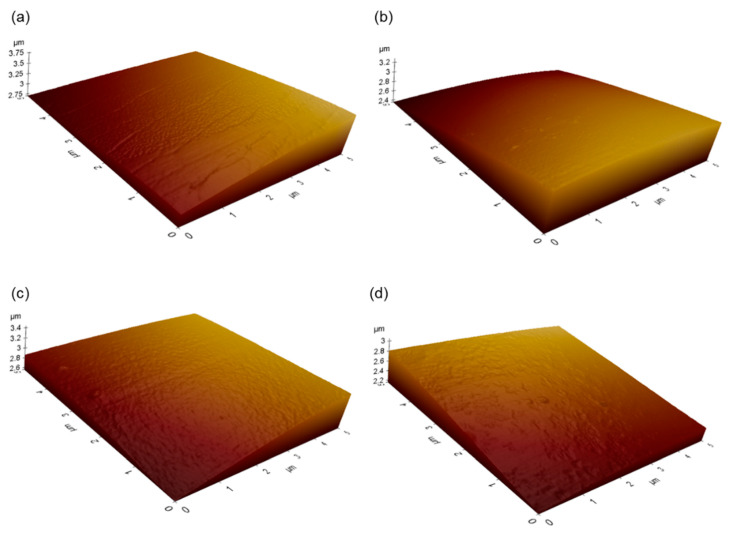
Atomic force microscopy images of EF and EA microspheres. Microspheres were prepared using either (**a**,**b**) EF or (**c**,**d**) EA. Before emulsification with a dispersed phase, an aqueous phase was doped with (left) 0 or (right) 2 mL of each solvent.

Spherical PLGA microspheres were successfully prepared following our solvent extraction technique using EF or EA (Figure 1). When microspheres are produced by a conventional solvent extraction process using EA, their surface tends to be severely deflated and wrinkled. Conversely, even though EF and EA microspheres reported in this study occasionally displayed mild dents on the surface, they had very smooth surface. Before SEM analysis, most samples are dried and sputtered with gold and exposed to an intensive beam of electrons. These invasive treatments may modify the micro-texture of PLGA microspheres. By comparison, AFM is recognized as a non-invasive tool that characterizes the morphology of PLGA microspheres without altering their original surface characteristics [18]. Therefore, AFM was used to evaluate 3D images of EF and EA microspheres prepared under various manufacturing conditions (Figure 2). Their AFM images agreed with the SEM micrographs shown in Figure 1, proving that our microencapsulation procedures led to the formation of non-porous microspheres with smooth surface. All EF and EA microspheres did not contain macropores and cavities on their surface. 

**Figure 3:**
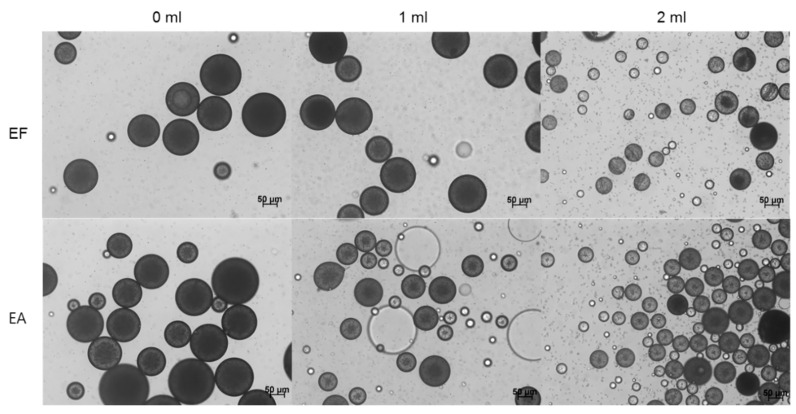
Light microscopy (LM) photographs of microsphere suspensions taken at the end of the primary solvent extraction step. A dispersed phase (250 mg PLGA/70 mg progesterone/3 mL EF or EA) was emulsified in an aqueous phase in which 0 to 2 mL of the solvent was pre-dissolved. The bar size is 50 μm.

**Figure 4:**
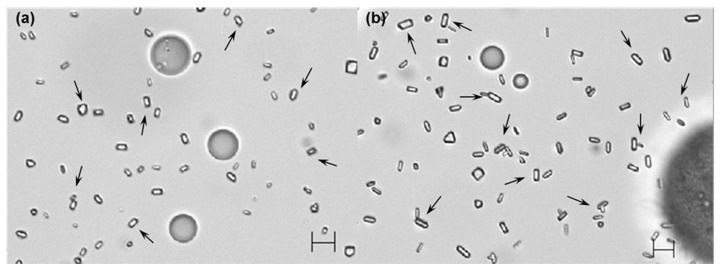
LM photographs of (**a**) EF and (**b**) EA microsphere suspensions. A dispersed phase (250 mg PLGA/70 mg progesterone/3 mL EF or EA) was emulsified in an aqueous phase containing 2 mL of the solvent. Arrows indicate progesterone microcrystals dispersed in the aqueous phases of the microsphere suspensions. The bar size is 10 μm.

Irrespective of which solvent was used for microencapsulation, one of the most critical process parameters of our preparative technique was the solvent amount pre-dissolved in an aqueous phase before emulsification. An optical microscope was used to investigate the status of a microsphere suspension at the end of the primary solvent extraction step described in the experimental section. When an organic solvent-free aqueous phase was engaged in emulsification with a dispersed phase, relatively large microspheres were present in the microsphere suspension, regardless of the solvent type (Figure 3). Especially in the EA-based microencapsulation process, fiber-like PLGA agglomerates were also produced (Figure S1). However, such a phenomenon did not occur in the practice of the EF-based microencapsulation process. It should also be noted that neither of EF and EA microsphere suspensions contained micron-sized progesterone crystals. Unlike the above results, adding EF or EA in an aqueous phase prior to emulsification led to the production of microsphere suspensions with very different characteristics. First, microspheres with significantly smaller sizes were produced (Figure 3). More importantly, numerous progesterone crystals appeared in microsphere suspensions. Figure 4 shows progesterone microcrystals dispersed in the aqueous phases of EF and EA microsphere suspensions. It was also worthwhile to note that the presence of extra EA in an aqueous helped suppress the formation of fiber-like PLGA agglomerates. 

**Figure 5:**
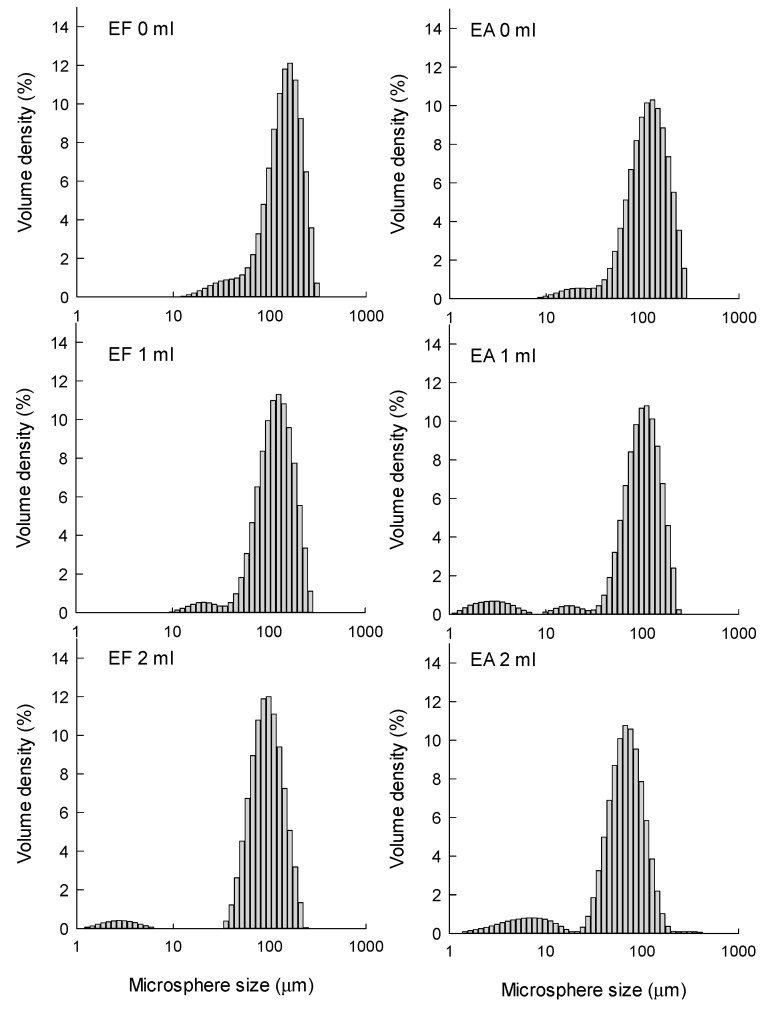
The dependence of microsphere size distribution upon solvent type and its pre-dissolved amount. PLGA (250 mg) and progesterone (70 mg) was dissolved in 3 mL of either EF or EA. The dispersed phase was emulsified in an aqueous phase containing 0 to 2 mL of each solvent.

The size distributions of EF and EA microspheres were measured at the end of the primary solvent extraction step (Figure 5). Under all manufacturing conditions, EF microspheres were slightly larger than EA microspheres, but they showed acceptable span indices indicative of size homogeneity. The size-reducing effect of the pre-dissolved extra solvent was clearly demonstrated in a quantitative manner (Table 2). For example, when the pre-dissolved EF volume varied from 0 to 2 mL, D_50%_ decreased from 152 to 97.5 μm. In cases of EA microspheres, their corresponding D_50%_ values were 122 and 69 μm, respectively. Figure 5 also demonstrates that particles smaller than 10 μm are present in the microsphere suspensions prepared using extra EF- and EA-containing aqueous phases. They are mainly progesterone crystals, as evidenced by light microscopy (LM) photographs shown in Figure 4. Therefore, the wet sieving step was implemented in our microencapsulation process in order to separate unentrapped progesterone crystals from EF and EA microsphere suspensions. As shown in LM photographs (Figure S2), our wet sieving was effective in removing unentrapped progesterone crystals from the microsphere suspensions. As a result, drug microcrystals were not found on the surface of EF and EA microspheres, as shown in SEM micrographs (Figure 1). 

**Figure 6:**
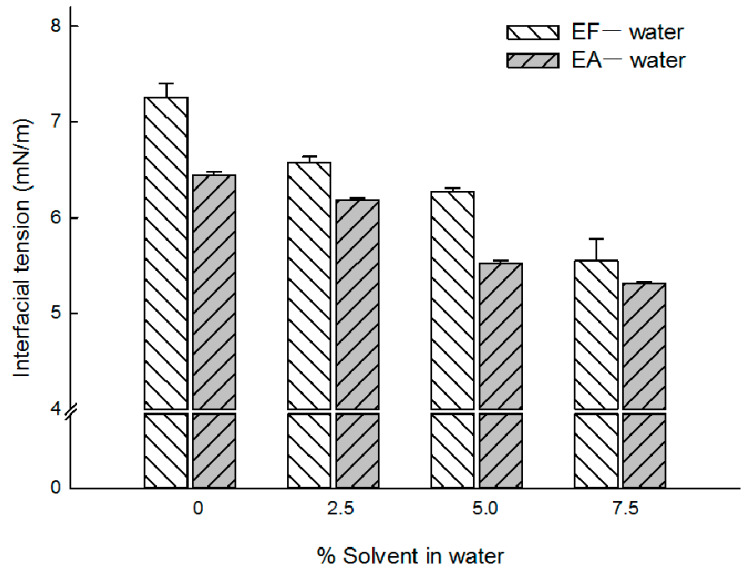
Effect of the aqueous solvent concentration upon the solvent−water interfacial tension. The pendant drop principle was utilized to calculate interfacial tension. There exists a proportional relationship between the aqueous solvent concentration and interfacial tension.

It was speculated that adding some extra solvent in an aqueous phase might decrease the organic solvent−aqueous phase interfacial tension. Changes in interfacial tension would have several ramifications in emulsification. The EA−water interfacial tension was measured to be 6.44 ± 0.04 mN/m (Figure 6). This value was quite similar to those values of 6.80 and 7.25 mN/m reported elsewhere [19,20,21]. The EF−water interfacial tension was determined to be 7.26 ± 0.14 mN/m. Increasing an aqueous solvent concentration resulted in a proportional decrease in interfacial tension, irrespective of the solvent type. These interfacial tension data might be useful to explain why the microsphere size decreased consistently with the aqueous solvent concentration increasing (Figure 1 and Figure 3, Table 2). 

**Figure 7:**
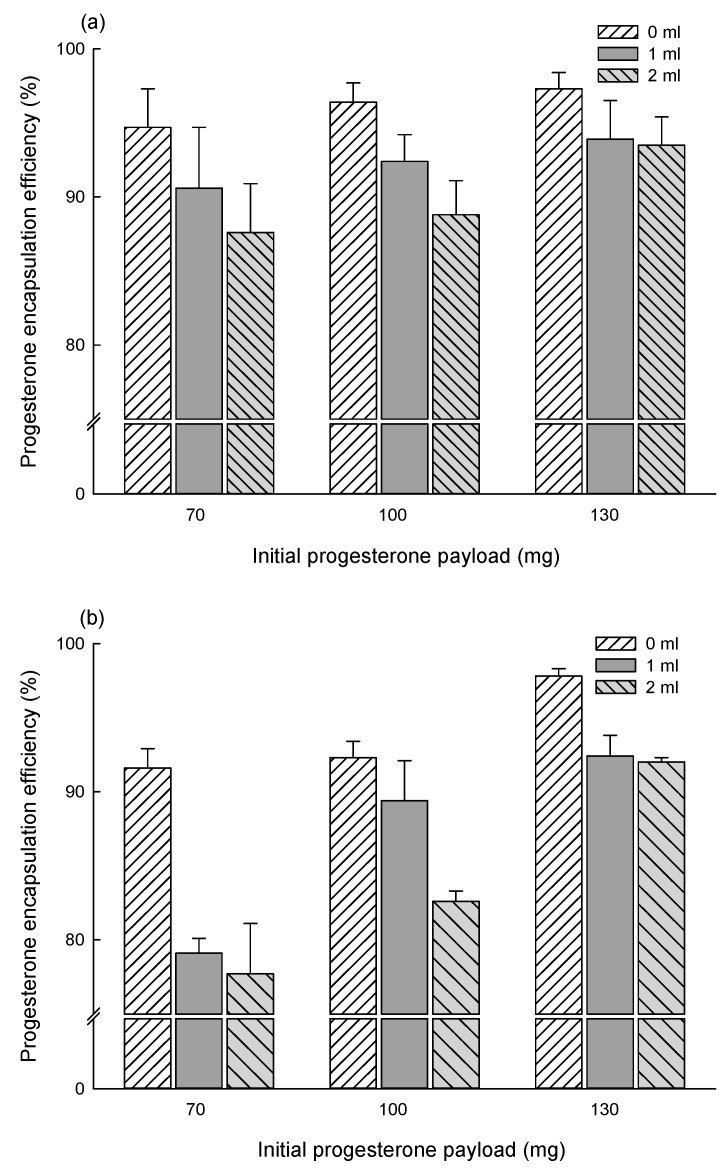
Effect of phase compositions upon progesterone EE. To produce microspheres, a PLGA/drug/solvent dispersed phase was emulsified in an aqueous continuous phase in which the solvent (0, 1, or 2 mL) was pre-dissolved before emulsification. The dispersed solvent used for microencapsulation was either (**a**) EF or (**b**) EA.

Figure 7 shows progesterone encapsulation efficiencies (EE) observed with various EF and EA microspheres. The EF-based microencapsulation gave excellent drug EE data. When microspheres were prepared by emulsifying a dispersed phase (70 mg progesterone, 250 mg PLGA, 3 mL EF) in an EF-free aqueous phase, progesterone EE was 94.7 ± 2.6%. Increasing the pre-dissolved EF volume to 1 and 2 mL, the drug EE was slightly reduced to 90.6 ± 4.1% and 87.6 ± 3.3%, respectively. When an initial progesterone payload was increased to 100 and 130 mg, the presence of extra EF in an aqueous phase also caused similar effects upon drug EE. When an EA-free aqueous phase and an initial progesterone payload of 70 mg were used for microencapsulation, the resultant drug EE was 91.6 ± 1.3%. Likewise, the case of the EF-based microencapsulation process, increasing its pre-dissolved amount to 1 and 2 mL caused reductions in its EE to 79.1 ± 1.0% and 77.7 ± 3.4%, respectively. The EF-based microencapsulation process always accompanied higher EE results than the EA-based one, except that 130 mg of progesterone was used for microencapsulation. Overall, progesterone EE was affected by the solvent type, its aqueous concentration, and initial drug payload. 

**Figure 8:**
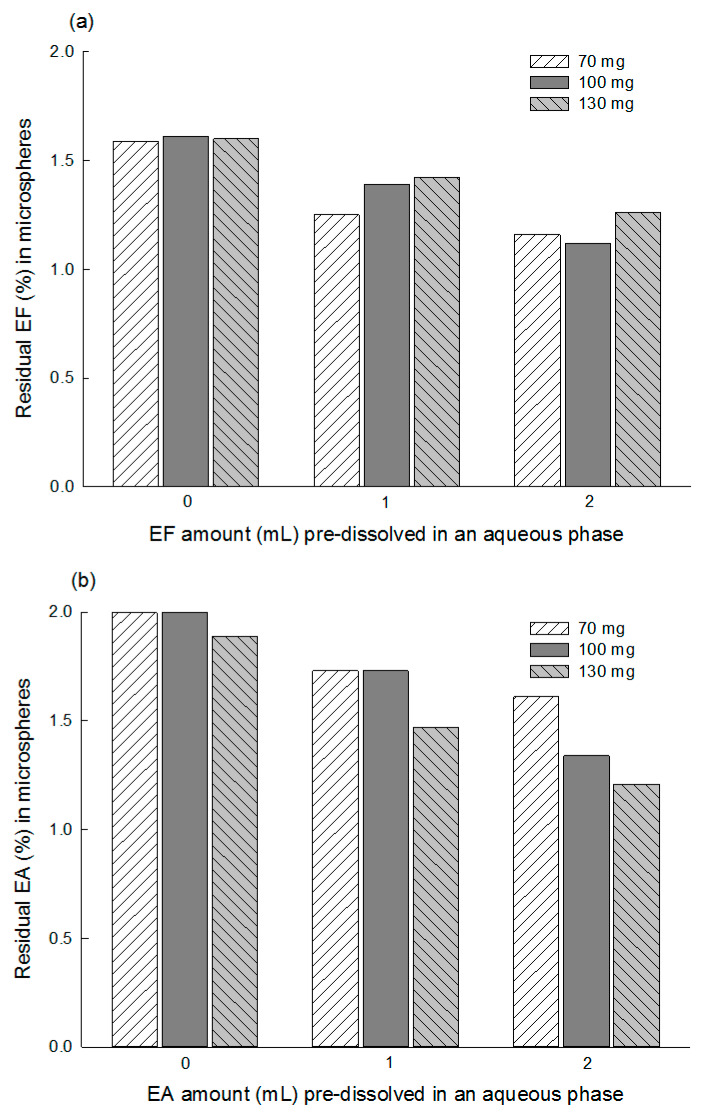
TGA thermograms showing residual (**a**) EF and (**b**) EA contents in microspheres. Their residual contents are affected by the solvent type and its pre-dissolvent amount in an aqueous phase prior to emulsification.

Residual solvent contents in EF and EA microspheres were determined by TGA (Figure 8). EF microspheres prepared using an EF-free aqueous phase contained 1.59%~1.61% of residual EF. Its residual solvent levels became lower upon the addition of extra EF in the aqueous phase prior to emulsification. Similar patterns were observed from EA microspheres, but their residual solvent levels were a little higher, compared to those of EF microspheres. To confirm the residual solvent amounts determined by TGA, GC experiments were also performed. Figure S3 shows a typical GC chromatogram illustrating the peaks of EF and EA. The residual EF content in the microspheres (250 mg PLGA/100 progesterone/0 mL extra EF) was measured to be 1.7 ± 0.1%. The residual EA in the corresponding microspheres was 2.2 ± 0.1%. These GC results are quite similar to the TGA data shown in Figure 8. 

**Figure 9:**
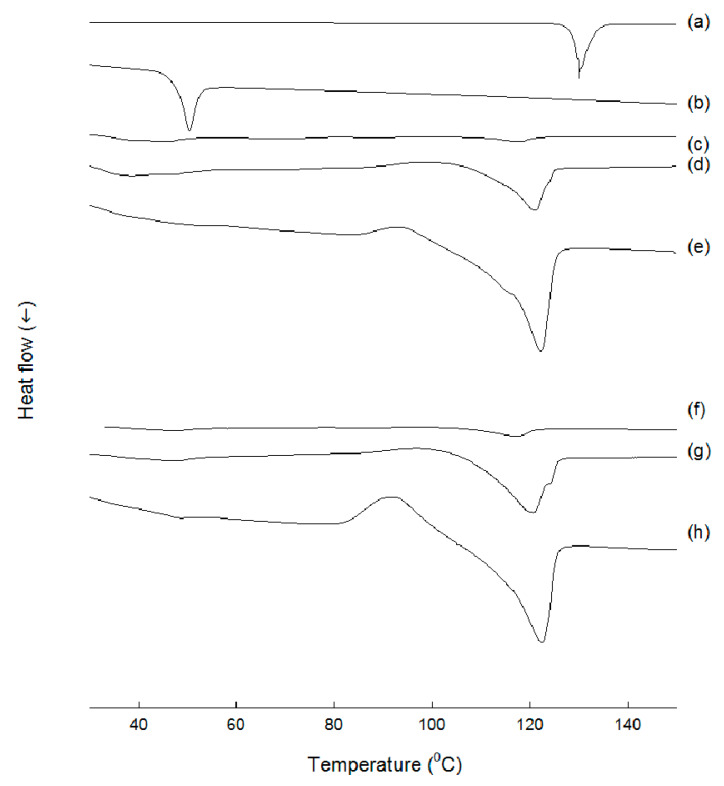
DSC thermograms of (**a**) progesterone, (**b**) PLGA as-received, (**c**~**e**) EF microspheres, and (**f**~**h**) EA microspheres. Actual progesterone payloads in the microspheres were (**c**) 20.7%, (**d**) 25.9%, (**e**) 30.0%, (**f**) 20.0%, (**g**) 22.6%, and (**h**) 26.6%.

DSC was used to measure thermal properties of various samples. Progesterone exhibited a sharp melting peak at 130 °C, while PLGA raw powders displayed a distinct Tg of 41 °C (Figure 9). When EF microspheres containing 20.7% progesterone was subject to a DSC analysis, the sharp melting point of progesterone almost vanished. This phenomenon indicated that progesterone was molecularly dispersed inside the matrix of the EF microspheres. In addition, the microspheres did not exhibit a distinct Tg. By contrast, EF microspheres with progesterone payloads of 25.9% and 30.0% exhibited a lower melting point for the drug. These results suggest that progesterone underwent polymorphic transition during microencapsulation. Thermal characteristics of EA microspheres were very similar to those of EF microspheres. 

**Figure 10:**
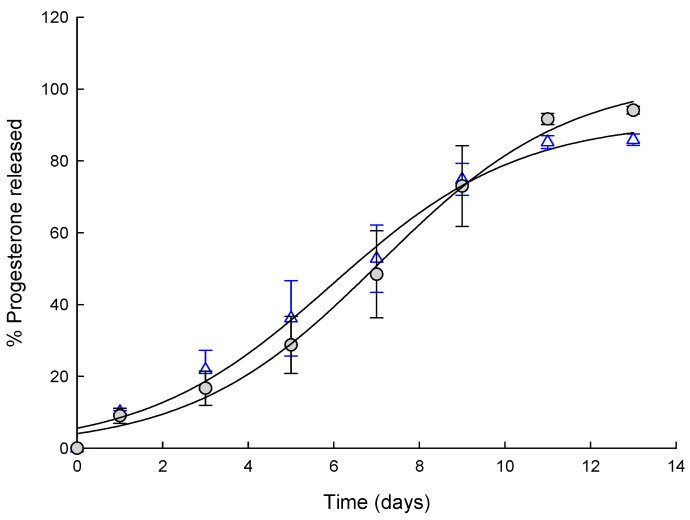
Dissolution profiles of progesterone observed with (○) EF microspheres and (△) EA ones.

Figure 10 shows progesterone release from EF and EA microspheres. The dissolution profiles of progesterone observed with both microspheres were similar to each other. Typically, drug release from PLGA microspheres is featured with a triphasic profile consisting of initial burst, subsequent lag phase with little release, and full-fledged release. In our experiments, both EF and EA microspheres did not show substantial burst and provided continuous progesterone release patterns. Our data are in line with the general perception that microspheres without any free drug crystals on their surface usually do not give burst effect. 

## 4. Discussion

### 4.1. Hansen Solubility Parameter (HSP) Distance

**Figure 11:**
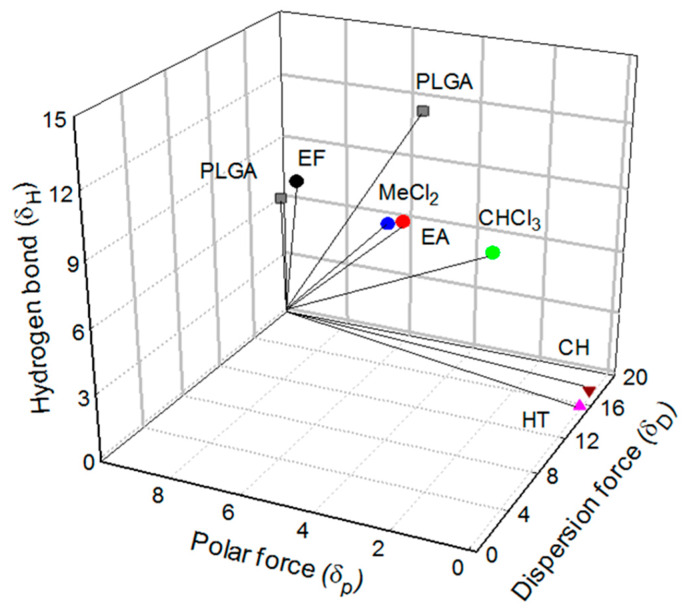
A three-dimensional plot standing for Hansen solubility parameters of PLGA 50:50, PLGA 75:25, methylene chloride (MeCl_2_), EF, EA, CHCl_3_, cyclohexane (CH), and *n*-heptane (HT).

Solubility parameters of PLGA and several solvents are shown in Figure 11. *δ_D_* is the energy density from dispersion bonds between molecules, and *δ_P_* is the energy from dipolar intermolecular force between molecules. *δ_H_* represents the energy from hydrogen bonds between molecules. As reported elsewhere [22,23], the solubility parameters of PLGA 50:50 are [16.38, 9.13, 7.12], and those of PLGA 75:25 are [18.7, 5.4, 11.6]. The following equation was used to calculate the distance between Hansen parameters of PLGA and each solvent:

Ra = {4 × (*δ_D_*_1_ − *δ_D_*_2_)^2^ + (*δ_P_*_1_ − *δ_P_*_2_)^2^ + (*δ_H_*_1_ − *δ_H_*_2_)^2^}^0.5^,

where subscript 1 serves as a sign of PLGA and subscript 2 stands for solvent. Ra is termed as the Hansen solubility parameter (HSP) distance that is an estimate of how compatible two molecules are. The smaller Ra is, the more likely they are alike. The Ra value of 8 is generally regarded as a good estimate between solvent and non-solvent for a polymer. Ra values between the two PLGA polymers and various organic solvents are calculated. 

PLGA 50:50 and PLGA 75:25 are insoluble in cyclohexane [16.8, 0, 0.2] (Ra = 22.9, 25.5) and heptane [15.3, 0, 0] (Ra = 23.3, 26.5). Those Ra values are far beyond 8, indicating that cyclohexane and heptane are antisolvents toward the two PLGAs. In fact, PLGA polymers are practically insoluble in both solvents. Methylene chloride [18.2, 6.3, 6.1], an excellent solvent for PLGA 50:50 and PLGA75:25, shows Ra values of 4.7 and 5.7. The corresponding Ra values of chloroform [17.8, 3.1, 5.7], being often used to prepare PLGA microspheres, are 6.8 and 6.6. The HSP distances between PLGAs and EF range from 2.3 to 7.8, whereas Ra values of PLGA−EA are 4.0 and 7.3. All these data support that EF serves as a good solvent for PLGA polymers. A recent paper lists a variety of solvents that can be used in the manufacturing process of PLGA microspheres and their characterization, but EF is not mentioned [16]. Our findings support that EF, possessing a number of advantages over conventional organic solvents, is an excellent solvent of choice that can be applied to microsphere manufacturing processes.

### 4.2. Limitations of Prior EF-Based Microencapsulation Processes

Previously, Sun et al. used EF to prepare poly(ε-caprolactone)/poly(ethylene glycol)/poly(ε-caprolactone) microspheres via an emulsion-based process [24]. They could not manufacture microspheres under their experimental conditions. Even though they did not elaborate on the reason, to our opinion, that was because they used a very high aqueous to dispersed phase volume ratio (150:3). Upon emulsification, EF in the dispersed phase might have quickly diffused into the water phase due its high water solubility. This might lead to the formation of polymeric agglomerates rather than emulsion droplets. A similar phenomenon was observed when a conventional EA-based microencapsulation process was employed to prepare microspheres. For instance, in the work of Li et al., fiber-like PLGA agglomerates also were present in the microspheres suspension [25]. The microencapsulation methods using organic solvents with considerable water solubility often face these obstacles. Especially at a high aqueous to dispersed phase volume ratio, mixing conditions (e.g., mixer type, mixing energy, and mixing rate) are also reportedly critical factors in determining the overall outcome in micro- and nano-particle formation [26]. On the contrary, in our microencapsulation process using EF and EA, the aqueous to dispersed phase volume ratio was purposely set at a relatively low aqueous to dispersed phase volume ratio of 25:3. Further, 1 or 2 mL of each solvent was pre-dissolved in the aqueous phase prior to emulsification. These strategies help suppress the diffusion of the solvent molecules in a dispersed phase into the aqueous phase. Accordingly, o/w emulsion droplets stabilized temporarily can be generated and then hardened into microspheres through solvent extraction. However, it should be cautioned that pre-dissolving too much extra organic solvent in an aqueous continuous phase increases the propensity for drug crystallization (Figure 3 and Figure 4). 

### 4.3. Solvent Effects upon Microsphere Morphology

The solvent effect upon the microsphere morphology is a frequently discussed topic. In general, PLGA microspheres prepared with methylene chloride display spherical morphology with smooth surface. On the contrary, an EA-based emulsion process leads to the formation of irregularly shaped microspheres with severely deflated and rough surface [25,27]. This phenomenon is exacerbated, especially when a small volume of a dispersed phase is emulsified in a large amount of an aqueous continuous phase. The greater polarity and water miscibility of EA, in comparison to methylene chloride, was held accountable for the formation of such microspheres [15,28]. When an EA- or EF-free aqueous phase was used in our microencapsulation process, some indentations on the microsphere surface were observed (Figure 1). However, the imperfection level of the microsphere surface was very mild. Additionally, spherical microspheres having very smooth surface could be produced by using an aqueous phase pre-dissolved with extra EF or EA prior to emulsification. 

### 4.4. Determinants of Drug Encapsulation Efficiency

There are several reports dealing with the influence of solvent type upon drug EE. Relevant conclusions are in dispute, but it is often reported that lower drug EE is attained with an EA-based microencapsulation method [15,28]. For example, when ibuprofen was loaded into PLGA microspheres via a methylene chloride-based process, its EE ranged from 23.2% to 79.8%, depending upon manufacturing conditions. When methylene chloride was replaced with EA, ibuprofen EE ranged from 16.7% to 58.2%. A similar conclusion was reached with somatostatin acetate [28]. It was reported elsewhere that the inclusion of EF in a dispersed phase had a detrimental effect on drug EE [29]. For instance, when an EF−methylene chloride mixture was used to encapsulate felodipine into microspheres, its EE was 56.6 ± 6.8%. When methylene chloride alone was used, the corresponding EE was up to 73.5 ± 4.7%. The authors drew a conclusion that the higher the water solubility of a dispersed solvent, the lower the drug EE. The relevance of interfacial tension to drug EE was also highlighted elsewhere [30]. A water-immiscible solvent exhibiting high interfacial tension (e.g., methylene chloride−water interfacial tension = 20.4 mN/m) was advantageous for improving drug EE. The work of Aragón et al. focused on the importance of solubility parameters [31]. They hypothesized that closer the values of solubility parameter of a dispersed solvent and a drug were, greater was the drug EE. However, it should be noted that the use of methylene chloride does not always bring high EE to many hydrophobic drugs. For example, when nor-β-lapachone was loaded into PLGA microspheres via a methylene chloride-based solvent evaporation process, its EE was only 19.4% [32]. 

Our microencapsulation processes using EF and EA, however, provide very high progesterone EE. Compared to methylene chloride, these solvents have much lower interfacial tension and greater water miscibility (Table 1, Figure 6). Furthermore, among the 3 organic solvents, EF has the highest water solubility. Despite these aspects, the EF-based microencapsulation process employing the aqueous to dispersed phase volume ratio of 25:3 was able to provide almost complete drug EE. When compared to EA, EF provided better progesterone EE in most cases. Our study emphasizes that interfacial tension, water miscibility, and solubility parameters are not absolute determinants of drug EE. Due attention should always be given to the importance of understanding and optimizing a microsphere manufacturing process. It is conceivable that fast solidification rates of emulsion droplets into microspheres contribute to achieve high drug EE [33,34]. Accordingly, our microencapsulation process is designed to generate emulsion droplets stabilized temporarily and to realize their quick transformation into solid microspheres, making use of their partial water miscibility. Under this condition, progesterone also precipitates quickly inside microsphere matrices, thereby providing excellent drug EE. 

It is also worth mentioning that, during typical emulsion-based microencapsulation processes, hydrophobic drugs tend to crystallize on the surface of microspheres and/or in the aqueous continuous phase. For example, drug crystals (e.g., clonazepam, levonorgestrel, curcumin derivative, ivermectin, estradiol, ibuprofen, and dexamethasone) formed when solvent removal was carried out at a constant rate [25,35,36,37,38,39,40]. Representative parameters affecting the propensity of drug crystallization include drug payload in a dispersed solvent, solvent type and composition, solvent removal rate, and aqueous emulsifier concentration. Several mechanisms have been proposed to elaborate on drug crystallization. For example, Birnhaum et al. hypothesized interfacial drug accumulation at a dispersed phase–aqueous phase that would lead to drug deposition on the microsphere surface [38]. It was proposed elsewhere that at high drug payloads, drug crystals would protrude to the microsphere surface [5]. Another speculation was that drug molecules diffused from emulsion droplets to an aqueous phase, thereby forming nuclei and crystals in the aqueous phase [39]. In our EA or EF microencapsulation processes using the solvent-free aqueous to organic phase volume ratio of 25:3, progesterone crystals do not appear in their microsphere suspensions (Figure 3). Under this condition, emulsion droplets quickly change into semisolid microspheres, due to the partial water miscibility of EF and EA. Progesterone remains entrapped inside the precipitated PLGA matrix, giving almost complete drug EE. As a result, progesterone microcrystals are not seen in the aqueous phases of the microsphere suspensions (Figure 3). On the contrary, pre-dissolving EF or EA in an aqueous phase prior to emulsification contributes to the formation of progesterone crystals in their microsphere suspensions (Figure 3 and Figure 4). It is likely that the pre-dissolved solvent in an aqueous solution helps generate emulsion droplets with much smaller sizes (Figure 3, Table 2). Furthermore, drug solubility in the solvent-containing aqueous phase is increased, and the hardening rate of emulsion droplets into microspheres is slowed down. Given that these situations lead to the enhancement of drug flux across emulsion droplets toward the aqueous phase, there exists a driving force for the formation of drug nuclei and subsequently crystals. As a result, the resultant EF and EA microspheres suffer minor reductions in progesterone EE (Figure 7).

## 5. Conclusions

Among non-halogenated dispersed solvents, ethyl acetate has been most commonly used for the manufacturing PLGA microspheres in the pharmaceutical industry. The present study demonstrates that ethyl formate, a non-halogenated ICH Class 3 solvent, is an interesting alternative that can be used in the preparative technique of drug-containing PLGA microspheres. Its use can help not only minimize the appearance of PLGA aggregates and unentrapped drug crystals during microencapsulation, but also produce PLGA microspheres with acceptable quality attributes. It would be meaningful to investigate in a future study how microsphere formulation (e.g., polymers of different types and molecular weights; drugs of varying hydrophobicity) and process variables affect the process capability of the ethyl formate-based microencapsulation technique. 

## Figures and Tables

**Table 1:** Physicochemical properties of ethyl formate and ethyl acetate that are ICH Class 3 solvents.

Property	Ethyl Formate *^a^*	Ethyl Acetate *^b^*
Formula	HCOOC_2_H_5_	CH_3_COOC_2_H_5_
Molecular mass (g/mol)	74.1	88.1
Boiling point (°C)	52−54	77
Density (g/cm^3^)	0.92	0.9
Solubility in water (g/100 mL)	10.5	8.7
Log P (octanol/water)	0.23	0.73

*^a^*http://www.inchem.org/documents/icsc/icsc/eics0623.htm; *^b^*http://www.inchem.org/documents/icsc/icsc/eics0367.htm.

**Table 2:** Dependence of the particle size distribution of a microsphere suspension upon solvent type and its pre-dissolved amount in an aqueous phase.

Solvent	Amount (mL)	D10%	D50%	D90%	Span
EF	0	72.2	152	243	1.12
EF	1	66.6	126	211	1.15
EF	2	55.3	97.5	162	1.1
EA	0	59.2	122	214	1.27
EA	1	41	103	176	1.31
EA	2	16.2	69	121	1.52

A dispersed phase (250 mg PLGA/70 mg progesterone/3 mL EF or EA) was emulsified in an aqueous phase containing 0, 1, or 2 mL of a solvent, and the resultant emulsion was transformed to a microsphere suspension. The size unit is μm.

## Supplementary Materials

The following are available online at https://www.mdpi.com/1999-4923/12/5/425/s1, Figure S1. LM photograph of PLGA agglomerates appearing in an EA microsphere suspension. To prepare microspheres, a dispersed phase (250 mg PLGA/70 mg progesterone/3 mL EA) was emulsified in an EA-free aqueous phase. When the emulsion was subject to solvent removal, not only embryonic microspheres but also fiber-like PLGA agglomerates were generated. The bar size is 500 μm. Figure S2. LM photographs of (top) EF and (bottom) EA microsphere suspensions before and after wet sieving. A dispersed phase (250 mg PLGA/70 mg progesterone/3 mL EF or EA) was emulsified in an aqueous phase in which 0 to 2 mL of the solvent was pre-dissolved. The bar size is 50 μm. Figure S3. A typical GC chromatogram of a microsphere sample that followed the analytical procedure described in text. Arrows on the gas chromatogram indicate the peaks of EF and EA.
