# Supplementary material for: Qualification of Non-Halogenated Organic Solvents Applied to Microsphere Manufacturing Process
Source: Pharmaceutics. 2020 May 6;12(5):425. doi: 10.3390/pharmaceutics12050425 (PMC7285046; doi:10.3390/pharmaceutics12050425)
Supplement: Supplementary file 1 [file pharmaceutics-12-00425-s001.pdf]

pharmaceutics

Supplementary Materials: Qualification of Non-Halogenated Organic Solvents Applied to Microsphere Manufacturing Process

Hyunjin Shim and Hongkee Sah *

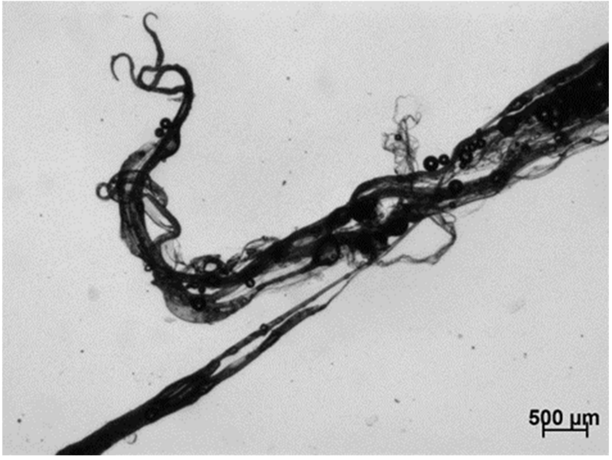

Figure S1. LM photograph of PLGA agglomerates appearing in an EA microsphere suspension. To prepare microspheres, a dispersed phase (250 mg PLGA/70 mg progesterone/3 ml EA) was emulsified in an EA-free aqueous phase. When the emulsion was subject to solvent removal, not only embryonic microspheres but also fiber-like PLGA agglomerates were generated. The bar size is 500 μm.

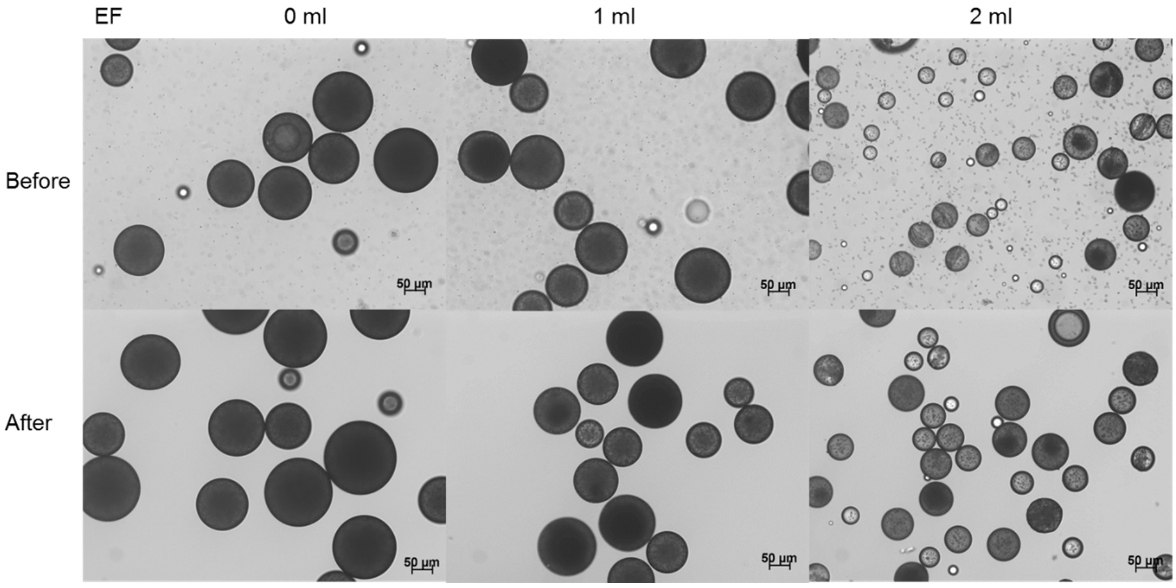

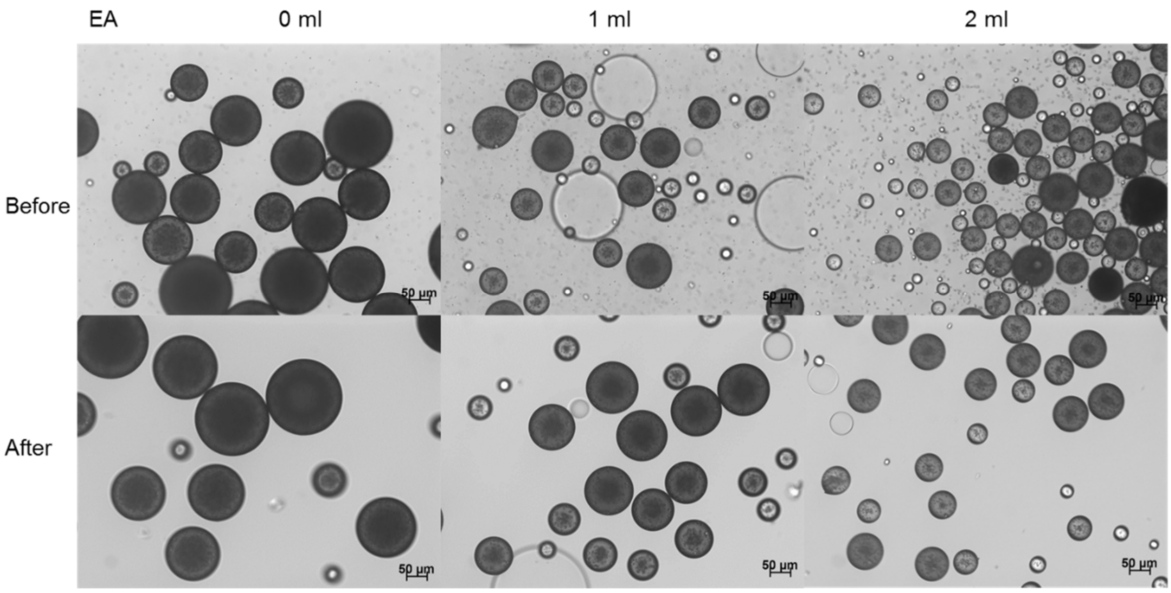

Figure S2. LM photographs of (top) EF and (bottom) EA microsphere suspensions before and after wet sieving. A dispersed phase (250 mg PLGA/70 mg progesterone/3 ml EF or EA) was emulsified in an aqueous phase in which 0 to 2 ml of the solvent was pre-dissolved. The bar size is 50 μm.

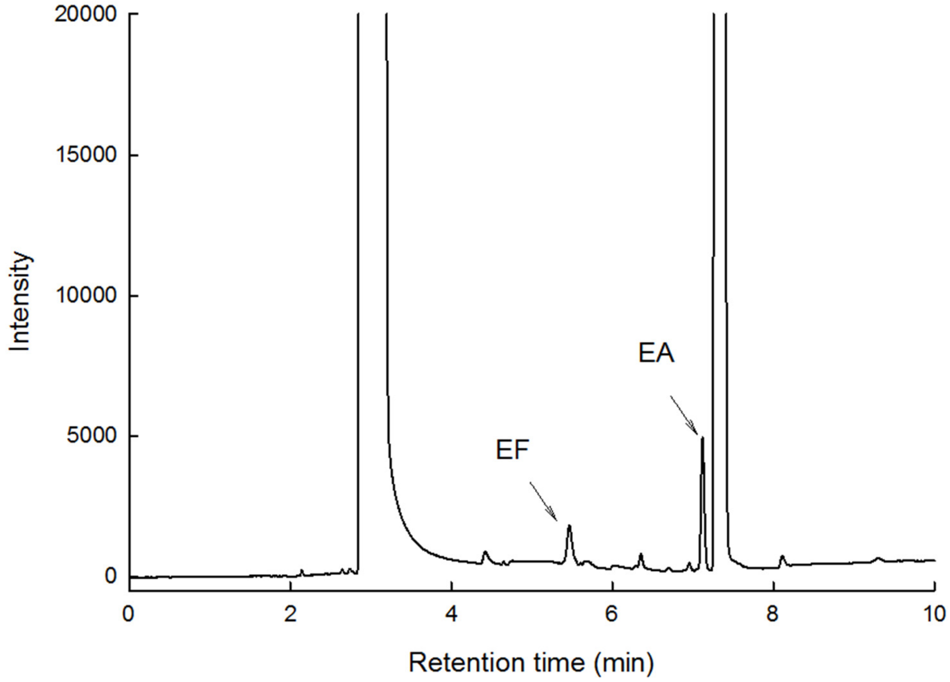

Figure S3. A typical GC chromatogram of a microsphere sample that followed the analytical procedure described in text. Arrows on the gas chromatogram indicate the peaks of EF and EA.
